# The Effectiveness of Statins as Potential Therapy for Multiple Sclerosis: A Systematic Review of Randomized Controlled trials

**DOI:** 10.7759/cureus.18092

**Published:** 2021-09-19

**Authors:** Mohammed A Abdalla, Christine M Zakhary, Hiam Rushdi, Jaafar A Hamdan, Kerolos N Youssef, Aafreen Khan, Safeera Khan

**Affiliations:** 1 Department of Clinical Pathology, Faculty of Medicine, Al-Neelain University, Khartoum, SDN; 2 Internal Medicine, California Institute of Behavioral Neurosciences & Psychology, Fairfield, USA; 3 Family Medicine, California Institute of Behavioral Neurosciences & Psychology, Fairfield, USA; 4 Medicine, American University of Antigua, St. John, ATG

**Keywords:** multiple sclerosis, statins, hydroxymethylglutaryl-coa reductase inhibitors, effectiveness, disease progression

## Abstract

Evidence of the effectiveness of statins, the lipid-lowering agents in retarding the progression of Multiple Sclerosis (MS), a disabling neurological disease with autoimmune etiology, have been highlighted in animal studies and observational studies. The proposed immune-modulatory actions and neuroprotective effects of statins make them a promising treatment option for MS that needs to be explored further. In this systematic review, we aim to investigate the role of different statins as monotherapy or in combination with the established MS medications in improving the clinical and radiological course of MS variants, including optic neuritis, using randomized controlled trials (RCTs). We systematically searched PubMed, PubMed Central (PMC), MEDLINE, Cochrane library, and Scopus databases using regular keywords and medical subject headings terms. Randomized controlled trials of any statin used in any variants of MS, including studies on statins used in optic neuritis published up to April 2021, were included in the review. Data on the effects on the relapse rate, the Expanded Disability Status Scale (EDSS) alterations, and the changes in Magnetic Resonance Imaging (MRI) lesions were collected from the included studies.

Seven studies with a total of 831 patients and an average duration of follow-up of six to 36 months were included in our review. Five trials were of statins add-on to interferon therapy in relapsing-remitting multiple sclerosis (RRMS), of which four studies were assessed to be of good quality while the remaining study featured a high risk of bias. One trial of simvastatin monotherapy in Secondary Progressive Multiple Sclerosis (SPMS) was included, which was assessed to be of good methodological quality with low risk of bias and adequate patient number. A trial of simvastatin monotherapy on patients with optic neuritis was included, which was evaluated as a good quality study. Still, it had a low number of participants and a short duration of follow-up.

We used the changes in disease relapse rate and EDSS as primary outcome variables and the MRI lesions changes as a secondary outcome variable. Studies in RRMS showed no significant effects on primary and secondary outcomes. The study on SPMS featured a significant improvement in EDSS in simvastatin-treated patients with no effect on relapse rate or MRI changes. Simvastatin use in optic neuritis enhances clinical visual outcomes with no significant effect on MRI changes or the rate of progression to definite MS.

In contrast to animal studies and observational studies, randomized controlled trials do not replicate the positive effects of statins used as monotherapy or combined with interferon beta in patients with RRMS and optic neuritis in relapse rate EDSS or MRI changes. However, trials of statins on SPMS showed a promising effect on disability progression (EDSS score), which might support the proposed immune-modulatory and neuroprotective role of statins and serve as a baseline for further RCTs applying statins as monotherapy or combination with other established MS therapies.

## Introduction and background

Multiple sclerosis (MS) is a chronic autoimmune demyelinating disease of the central nervous system (CNS), which is a leading cause of non-trauma-related disability, with an estimated disease prevalence of 2.5 million individuals worldwide [1]. It shows a female preponderance, with females being three folds more affected in comparison to males.

MS can be classified into one of the following subtypes according to the disease course and the tendency for relapse: Relapsing-remitting multiple sclerosis (RRMS) (70-80%), primary progressive multiple sclerosis (PPMS) (15-20%), secondary progressive multiple sclerosis (SPMS), along with progressive relapsing multiple sclerosis (PRMS) (5%), and some classes add Clinically Isolated Syndromes (CIS) including optic neuritis [2].

Hence up-to-date, there is no curative treatment for MS, the main target of the current MS therapeutics is to slow down the progression of the disease and reduce the frequency of relapses. Some of the drawbacks of the current first-line MS therapies are the variable effectiveness seen in some patients (could be explained by the complex pathology of the disease), the side effects profile of the medications, and the route of administration (most of the first-line medications are injectable), along with the issue of affordability [3]. Because of the limitations mentioned above of the first-line MS therapies, further studies searching for effective alternative medications with fewer side effects and comparable efficacy are warranted.

Statins, the 3-hydroxy-3-methyl glutaryl coenzyme A (HMG-CoA) reductase inhibitors, are well-established oral medications for hypercholesterolemia with reasonable safety and tolerability [4]. Evidence from in vivo animal studies of experimental autoimmune encephalomyelitis (EAE) revealed anti-inflammatory and pleiotropic immune-modulatory effects of statins; moreover, it reduces the first relapse rate of EAE [5-7]. Additionally, in vitro studies on peripheral immune cells extracted from individuals affected with MS demonstrates that statins have multiple anti-inflammatory effects [8-10]. These results suggest a promising role for statins in treating multiple sclerosis and served as a scientific base for research on the effectiveness of statins in MS.

**Figure 1:**
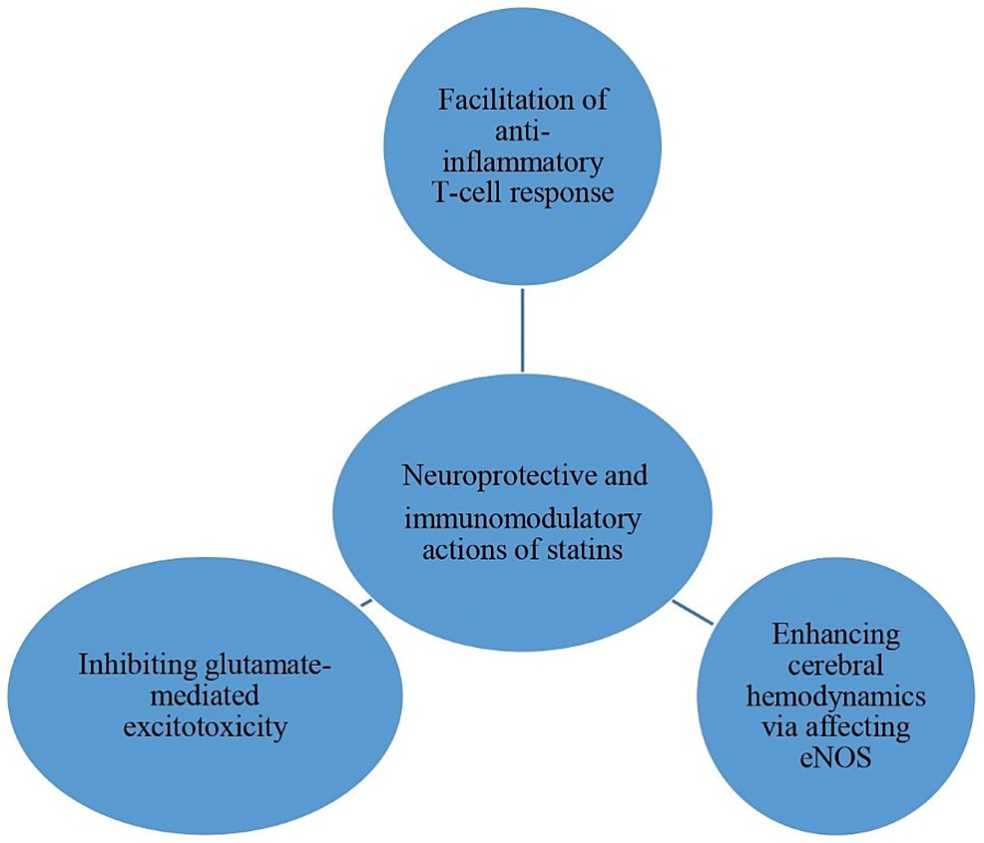
The potential mechanisms of statins effects in MS eNOS: endothelial nitric oxide

Studies suggest that the effects of statins in MS courses could be due to the facilitation of anti-inflammatory T-cell response, enhancing cerebral hemodynamics by altering the balance of nitric oxide production by endothelial cells (eNOS), and inhibiting glutamate-mediated excitotoxicity [7,11,12]. Figure 1 explains the potential mechanism of statins effects in MS.

The studies of statins used for MS treatment from human studies have expressed variable outcomes on the disease course. Three open-label studies in patients with RRMS demonstrated positive effects on MRI lesions number and the size of the MRI gadolinium-enhancing lesions (GEL) [13-15]; however, the subsequent larger-scale RCTs of statins, mainly applying simvastatin or atorvastatin monotherapy or co-therapy with established MS medications, have failed to demonstrate the effects suggested by the previous open-label studies; some showed negative effects on disease progression [16]. The purpose of this systematic review is to explore any possible evidence of the effect of statins used alone or combined with first-line MS therapies on the progression of the different subtypes of MS from RCTs.

## Review

Methods

This systematic review was conducted following the updated Preferred Reporting Items for Systematic Review and Meta-Analyses (PRISMA) 2020 guidelines [17].

Data Search Strategy

We conducted our data search for articles indexed in PubMed, PubMed Central PMC, MEDLINE (National Library of Medicine), Scopus, and Cochrane library from March 20 to April 15, 2021. A search strategy combining regular search terms and the Medical Subject Heading terms (MeSH) for studies indexed in PubMed, PMC, and MEDLINE was applied. For articles indexed in Scopus and Cochrane library, regular keyways were used for the search strategy. Table 1 summarizes our search strategy and the databases used.

**Table 1:** The data search strategy used for the review

Databases	Search strategy	Search results
PubMed, PubMed Central PMC, MEDLINE	("Multiple Sclerosis/therapy"[Mesh] OR Disseminated Sclerosis OR MS (Multiple Sclerosis)) AND ("Hydroxymethylglutaryl-CoA Reductase Inhibitors/therapeutic use"[Majr] OR Statins OR Statin OR HMG-CoA Reductase Inhibitor)) AND( ("Disease Progression"[Majr]) OR (Disease progression ) OR (disease outcome)OR (clinical outcome))	68
Scopus	“Multiple Sclerosis” AND Statins AND “Disease Progression”	585
Cochrane Library	Multiple Sclerosis AND Statins	1

Study Selection Eligibility 

The titles and abstracts of the research articles collected by the search strategy were independently checked by the first and the second authors, MA & CM. Any conflict about eligibility was discussed to reach an agreement. The relevant research papers were subsequently had their full texts checked to identify the articles that satisfy the selection criteria. We included the English language-published studies that involved statins monotherapy or in combination with other established MS therapies in patients with any type of MS, including optic neuritis. MS should be defined based on the Poser criteria [18], the original McDonald [19], or the revised McDonald criteria [20].

Bias Assessment and Quality check

All eligible research articles were then independently assessed for risk of bias by the first and the second authors. The quality of the eligible RCTs was checked using Version 2 of the Cochrane Risk-of-Bias Tool [21].

A Study was included in our systematic review if the bias assessment score was not classified as high in half or more of the assessment domains of the Cochrane Bias Assessment Tool. Each of the studies was evaluated for the risk of bias in the following domains: random sequence generation (selection bias), allocation concealment (selection bias), blinding (performance bias), detection bias, along with selective reporting (reporting bias), incomplete outcome data (attrition bias) and other risks of bias. Table 2 illustrates the result of the quality assessment for the studies.

**Table 2:** Summary of the quality check for the eligible studies (+): Low risk of bias, (?): Unclear risk of bias, (–): high risk of bias

Author (Year)	Random sequence generation (Selection bias)	Allocation concealment (selection bias)	Blinding (performance bias)	detection bias	Selective reporting (reporting bias)	Other bias	Incomplete outcome data (attrition bias)
Birnbaum et al (2008) [22]	?	+	+	+	+	?	+
Lanzillo et al. (2010)[23]	?	?	–	?	?	?	–
Sorensen et al. (2011) [24]	+	+	+	+	+	+	–
Tsakiri et al. (2012) [25]	+	+	+	+	+	?	–
Chataway et al (2014) [26]	+	?	+	+	+	+	+
Lanzillo et al. (2015)[27]	+	+	+	+	+	+	?
Ghasami et al. (2016) [28]	+	?	+	?	?	?	+

Outcomes Assessment

For studies on patients with MS, we used the clinical relapse rate and the alteration of disability status defined by the Expanded Disability Status Scale (EDSS) as primary outcome variables [29]. The level of MRI lesion change either in number or volume was used as a secondary outcome variable. Data about the changes in relapse rate, the EDSS, and MRI lesions changes related to statin use were extracted from the participating RCTs and presented in our review. Alteration in clinical visual outcomes, MRI lesion changes, and the conversion rate to MS were extracted and presented as outcome variables for patients with optic neuritis.

Results

**Figure 2:**
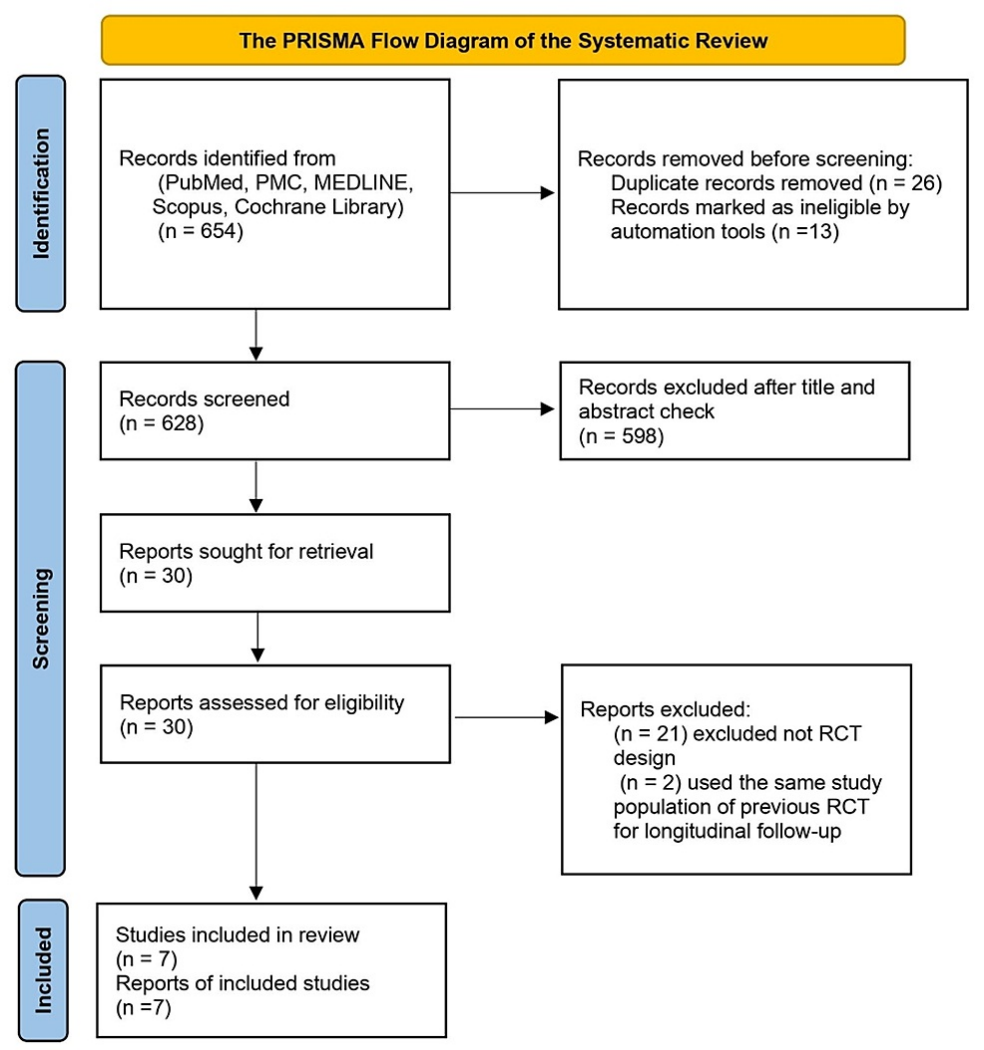
PRISMA flow chart for the systematic review PRISMA: Preferred Reporting Items for Systematic Review and Meta-Analyses

A total of 654 articles were identified by data search of the databases using a combination of regular keywords and Medical Subject Headings (MeSH) terms. Twenty-six duplicate studies were then removed using Microsoft Excel 2013 software. Out of the remaining 628 papers, 22 articles remained after titles & abstracts checking along with the application of automated inclusion and exclusion criteria. The full papers of the 22 studies were subsequently assessed for the inclusion and exclusion criteria. Finally, seven studies satisfied eligibility and quality check criteria. Figure 2 illustrates the steps followed in the conduction of this systematic review using the PRISMA 2020 flow chart [17].

Included Studies

A total of seven randomized control trials were included in our review. Five of the finalized studies were conducted on patients with relapsing-remitting Multiple Sclerosis, as defined by Poser, McDonald, or revised McDonald criteria [18-20]. The study by Chataway et al. evaluated the use of statins in Secondary Progressive Multiple Sclerosis. In contrast, Tsakiri et al. presented a trial of simvastatin monotherapy in patients with optic neuritis. No clinical trials were found on patients with primary progressive or progressive relapsing variants of MS [22-28].

Five trials evaluated the use of statins (atorvastatin or simvastatin) as add-on therapy to interferon beta [22-24,27,28]. In comparison, the remaining two trials examined statins as monotherapy for Multiple Sclerosis or optic neuritis [25,26]. A total of 831 patients were included in our review, of whom 627 patients had the relapsing-remitting disease, 140 patients had secondary progressive disease, and 64 patients were diagnosed with optic neuritis.

Trials on Relapsing-Remitting Multiple Sclerosis (RRMS)

Birnbaum et al. conducted a nine-month-follow-up-double blinded randomized controlled trial on 26 clinically stable patients with relapsing and remitting MS as defined by Poser [18], or McDonald criteria [19], who were on a high dose of subcutaneous interferon beta 1-a therapy for 0.5 to 3 years duration [22]. Seventeen patients were then randomized to receive interferon beta 1-a (44 micrograms) three times weekly plus atorvastatin 40 or 80 mg once daily. The other nine patients received interferon three times weekly added to a placebo for a total duration of six months. The trial population has no statistically significant difference in patient’s age, baseline EDSS, time on interferon therapy, course of the disease, MRI enhanced lesions, and several relapses on interferon.

The study carried a risk of selection bias as the process of patients’ allocation and concealment was not described in the study; additionally, the role of funding bodies in the conduction of the trial was not adequately explained, which might lead to a conflict of interest. However, the study had a low risk of bias in performance, detection, attrition, reporting, and detection processes.

Lanzilo et al. carried out the ACTIVE study, a 24-months-follow-up-open-label controlled trial of atorvastatin 20 mg added on to interferon beta 44 micrograms subcutaneously three times per week versus administration of interferon beta alone [23]. A total of 45 patients with relapsing and remitting MS as defined per McDonald criteria [19], who showed an inadequate response to initial interferon therapy, were involved in the trial with 21 patients allocated to atorvastatin daily with interferon beta-1-a three times weekly, and 24 patients received interferon 1-a three times per week monotherapy, following an initial 12 months of run through interferon therapy [23].

Recruited patients in the ACTIVE study were 18-50 years of age, who have at least one clinical or radiological evidence of disease activity on interferon therapy and did not receive any prior statin, immune-suppressant, or immune-modulator therapy. All the study participants were of comparable demographic, disease duration and outcome variables assessed. The study carries a high risk of performance and attrition bias caused by inadequate blinding and unbalanced drop-out rates between the study groups. The risk of selection bias was difficult to assess as the study did not report the randomization and concealment process details. Finally, the role of the funding company in the study conduct was not detailed, raising concerns about conflict of interest [23].

Sorensen et al. presented a multicenter double-blinded placebo-controlled trial of simvastatin 80 mg daily treatment added to intramuscular injection of interferon beta-1a versus placebo added to interferon. A total of 307 treatment naïve patients participated with RRMS as per Poser [18] or revised McDonald criteria [20] with baseline EDSS ≤5.5 and age between 18 and 55 years. Patients were allocated to receive simvastatin 80 mg with interferon beta (n: 151) or interferon with placebo (n: 155). The candidates were subsequently followed up for a duration of one to three years. The trial is of good quality; it expressed a detailed description of randomization, allocation concealment, and blinding process, therefore, it was classified as low risk of selection and performance bias. On the other hand, the study had a higher drop-out rate than expected on study design, putting it at high risk for attrition bias. The study was evaluated as low risk of other sources of bias; hence, it expressed the role of the funding company, which did not report to have apparent input to study design or conduct [24].

Lanzilo et al. presented the ARIANNA study, which is a 24 months follow-up trial of a double-blinded placebo-controlled design. The study involved a total of 154 patients with RRMS within five years of diagnosis who were on interferon beta 1-b therapy for a three to 12 months period. Enrolled patients' ages were between 18-50 years, and their average EDSS was between 1 and 5.5. The participants were randomized to receive either atorvastatin 20 mg per day added to interferon beta 1-b 8 MIU (million international units) subcutaneous injection once per day (n: 75) or placebo added to interferon (n: 79) [27]. Although the study method showed a low risk of selection, performance, reporting, and detection bias, there was a high risk of attrition bias caused by the high drop-out featured in the study.

Ghasami et al. conducted a randomized controlled trial on 95 patients with active RRMS as defined by modified McDonald criteria for an 18 Months duration of follow-up. Patients were allocated to receive atorvastatin 40 mg in addition to interferon beta intramuscular (IM) injection once weekly intervention (n: 50) or interferon beta intramuscular injection once-weekly monotherapy control (n: 45) [28]. Out of the recruited patients, most had at least one relapse over the 12 months before the study, or a minimum of three lesions on brain MRI, or both. Baseline EDSS was between 0-3.5, and the age of the patients involved was between 18 and 60 years of age. Patients in the intervention and control groups were comparable in baseline neurological variables at the beginning of the study [28].

The trial quality assessment revealed that it is of intermediate quality; the allocation method using random tables was mentioned in the study. Both the patients and the assessors were blinded; however, the details of allocation concealment were not mentioned in the study article. The study featured a high drop-out rate (32 patients); the study did not mention how this high drop-out was dealt with in the stage of data analysis and reporting. Overall, the study was classified as low risk of selection, performance, detection bias, and high attrition bias. There was not enough data to judge the risk of selective reporting and other sources of bias in the trial [28].

Trials on Secondary Progressive Multiple Sclerosis (SPMS)

Chataway et al. conducted a phase II multicenter double-blinded placebo-controlled trial on a total of 140 patients with Secondary Progressive Multiple Sclerosis (SPMS) for 24 months follow-up period. The study recruited 140 patients with SPMS as defined by revised McDonald criteria, who did not receive recent steroids or other immune-modulator or immunosuppressive agents. Recruited patients were of 18-65 years of age with baseline EDSS scores between four and 6.5. Seventy patients were allocated to receive simvastatin 80 mg daily, while the other 70 patients received a single dose of placebo daily. The patient groups were comparable for baseline characteristics at the beginning of the study [18,26]. The trial quality was good, and a low risk of bias was reported using Cochrane bias assessment tool domains. The trial manuscript explained the details of randomization generation, concealment, and blinding; furthermore, it adequately reported outcome variables and had a low and balanced drop-out rate that classified the trial as low risk of bias in all reported domains.

Trials on Optic Neuritis

Tasakiri et al. examined the role of simvastatin 80 mg on patients with optic neuritis in a six-month double-blinded placebo-controlled trial [25]. The study population was 64 patients with idiopathic optic neuritis who were randomized to a simvastatin arm or a placebo arm. Both medications were given once daily for the duration of the study. Patients were 16 to 59 years of age who had a first episode of optic neuritis on the affected eye, who were not on steroids or recent immunosuppressant with symptoms duration less than four weeks from the onset with reported reduced contrast sensitivity. The trial is classified as low risk of bias with good methodology quality. The randomization process was done using the computerized randomization method. The allocation was adequately concealed and sealed, containing randomization code was used, and both the patients and outcome assessors were blinded. No evidence of selective reporting-related bias was detected. However, the study had unbalanced drop-out butting some risk of attrition bias [25].

Intervention outcomes

To evaluate the effect of statins on MS progression, we analyzed the results from the trials involved in the review using the pre-specified outcome variables as surrogates for disease progression. We used disease relapse and EDSS as primary outcomes, while MRI changes were used as secondary outcomes for our analysis.

Primary Outcomes of Statin

Disease relapse: Disease relapse was a reported outcome in six of the seven participating randomized trials. Four studies were on RRMS patients, out of which three evaluated statins add-on to interferon on RRMS, and one study evaluated statin add-on to interferon on SPMS patients [22-24,26,27].

Two studies, by Birnbaum et al. [22] and Lanzilo et al. [23], on RRMS reported a significant difference in relapse rate. Lanzilo et al. concluded a lower rate of relapse per year of follow-up among statin and interferon combination group compared to interferon only group, on the contrary [23]. Birnbaum et al. reported a significantly higher rate of relapse (using combined MRI and clinical features) in the statin & interferon group compared to interferon monotherapy [22]. Sorensen et al. and the ARIANNA study demonstrated no significant difference in relapse rate, which used simvastatin, or atorvastatin combined with interferon, respectively, compared to placebo & interferon combination in RRMS [24,27].

Chataway et al., which examined simvastatin monotherapy in patients with Secondary Progressive Multiple Sclerosis SPMS, found no significant difference in relapse rate between simvastatin and placebo-treated patients at 24 months follow-up [26].

Modification of the Expanded Disability Status Scale (EDSS): EDSS was used for reporting intervention outcomes in five studies included in our review. Four studies expressed EDSS in patients with RRMS; Lanzilo et al. ACTIVE study reported EDSS score at 24 months of follow-up, Sorensen et al. measured mean EDSS at 12 and 36 months, while Ghasami et al. expressed EDSS difference between baseline and 18 months of follow-up [23,24,28]. The ARIANNA study used the proportion of patients without a sustained increase in EDSS at 6-24 months. For the SPMS trial, Chataway and co-authors presented the difference in EDSS at 24 months between simvastatin and placebo groups [26,27].

EDSS evaluation in trials on RRMS patients revealed no significant difference in EDSS score by Sorensen et al., Lanzilo et al. (ARIANNA), and Ghasami et al. study [24,27,28]. Lanzilo et al., in the ACTIVE study, showed a non-significant trend toward EDSS worsening at 24 months of follow-up in the interferon monotherapy arm compared to the atorvastatin treated group [23]. In patients with SPMS, Chataway et al. documented a significantly positive effect on EDSS in the simvastatin treated group compared to placebo [28].

Secondary Outcomes of Statins 

The change in the MRI lesions number or volume: The change in the MRI lesions was a reported outcome in six of the studies included in our review; all the six trials reported changes in MRI lesions number as one of their outcome variables; however, only Sorensen et al. and Chataway et al. examined the change in MRI lesion volume as one of its outcomes [24,26].

Four trials expressed no significant difference in MRI changes. Sorensen et al. and Chataway et al. reported non-significant differences in MRI lesion number and volume in intervention and treatment groups [24,26]. Additionally, Lanzilo et al. ARIANNA study and Ghasami et al. study reported no significant difference in MRI lesions number [27,28].

However, Birnbaum et al. and Lanzilo et al.'s ACTIVE study featured significant differences in MRI lesions between trial groups [22,23]. Lanzilo et al. reported fewer MRI lesions in statin interferon combination than interferon alone; on the other side, Birnbaum et al. detected a higher risk of MRI activity in the atorvastatin group [22, 23].

Outcomes of the study on statin effectiveness in optic neuritis as a CIS: Tsakiri et al. the only study found in patients with optic neuritis compared simvastatin 80 mg once per day monotherapy with placebo in a 6-month follow-up trial. The trial detected a significant difference between groups in visual acuity and color perception, a similar improvement in visual evoked potential (VEP) latency and VEP amplitude for the simvastatin group. However, the study had detailed no statistically significant difference in the total number of brain MRI lesions. The conversion rate to MS was not different between study groups [25].

Discussion

Statins used commonly as a treatment for hyperlipidemia and primary and secondary prevention of cardiovascular diseases showed anti-inflammatory, immune-modulatory, and neuroprotective actions that make it a promising treatment option in MS. The role of statins in multiple sclerosis was explored in animal studies which suggested a positive effect of statins on disease progression in the mouse model of MS. Although observational and open-label studies have detected beneficial effects on disease progression and MRI changes, these effects were not replicated in controlled trials.

Two previous systematic reviews explored the role of statins in MS in randomized controlled trials. Both concluded no adequate evidence of statins' effectiveness in retarding disease progression, relapse rate, EDSS, or improving the MRI changes in relapsing-remitting MS [3,16]. However, Pihl-Jensen et al.'s review suggested a positive effect of high dose simvastatin in the secondary progressive variant of MS along with the beneficial effect on disease course in patients with optic neuritis, which warrants further evaluation [3].

This systematic review aimed to explore the effectiveness of statins in improving disease progression in patients with multiple sclerosis measured by changes in clinical or radiological outcomes of the disease. Our review summarizes the result of seven randomized controlled trials with a total population of 831 patients and a duration of follow-up ranging between six months to 36 months. Table 3 summarizes the main features of the RCTs included in this systematic review.

**Table 3:** Summary of the main characteristics of the RCTs included in the review N: Total study population; I: intervention group; C: control group; RRMS: Relapsing-Remitting Multiple Sclerosis; SPMS: Secondary Progressive Multiple Sclerosis; EDSS:  Expanded Disability Status Scale; IFN: Interferon; MRI: Magnetic Resonance Imaging; SC: Subcutaneous; IM: Intramuscular.

Author (year)	Participants’ criteria	Duration of follow-up	Method	Participants	Intervention	Outcome of the intervention
Ghasami [28] (2016)	RRMS has one or more relapses, more than 3 spinal MRI lesions. EDSS: 0-3.5. Age: 18-60 years	18 months	Double-blind Randomized controlled trial	N (95) I (50) C (45)	Atorvastatin 40 mg + IM IFN	Relapse: not reported EDSS: No difference MRI lesions number: No difference
Lanzilo [27] (2015)	RRMS patients within 5 years from diagnosis. On IFN for 3-12 months. EDSS: 1-5.5. Age: 18-50	24 months	Double-Blind Randomized Placebo-Controlled Study	N (154) I (75) C (79)	Atorvastatin 40 mg + IFN b-1b SC Daily.	Relapse: no difference EDSS: No difference MRI lesions: no difference in lesions number
Chataway [26] (2014)	SPMS. Age: 18-65 years. EDSS: 4-6.5	24 months	Multicenter Double-blind placebo-controlled phase II Trials.	N (140) I (70) C (70)	Simvastatin 80 mg	Relapse rate: No difference EDSS : Improved EDSS MRI lesions : No difference
Tsakiri [25] (2012)	Diagnosed at Age 18 - 59 years. Symptom duration of <4 weeks	Six months	Placebo-controlled trial	N (64) I (32) C (32)	Simvastatin 80 mg	Relapse: No difference EDSS: Not reported MRI lesions: No difference
Sorensen [24] (2011)	RRMS. EDSS≤5.5. Age: 18 -55	range from 12-36 months	Multicenter double-blind placebo-controlled trial.	N: 307 I: (151) C: (156)	Simvastatin 80 mg + IFN beta-1a	Relapse rate: No difference EDSS : No difference MRI: no difference in lesions volume or number
Lanzillo [23] (2010)	RRMS. Age : 18- 50 years	24 months follow-up	Randomized controlled trial	N: (45) I: (21) C: (24)	Atorvastatin 20 mg + IFN beta-1-a	Relapse: Lower relapse rate in the treatment group EDSS: a non-significant trend toward improvement MRI lesions: less MRI lesions
Birnbaum [22] (2008)	RRMS. On high dose SC IFN-1a. Age: 16-68 years	6 months follow-up	Placebo-controlled	N: (26) I: (17) C: (9)	Atorvastatin 40 mg Atorvastatin 80 mg + IFN beta-1 a	Relapse: Higher rate of relapse EDSS: Not Reported MRI lesions: Worsened

Five of the studies covered the role of statins combined with interferon in patients diagnosed with RRMS at various stages of disease progression [22-24,27,28]. Three studies applied atorvastatin (20, 40, 80 mg) combined with subcutaneous interferon beta; out of these, one study examined atorvastatin 40 mg in combination with intramuscular interferon beta formulation, and the other one explored the effect of adding simvastatin 80 mg to intramuscular interferon beta. Four studies were of good quality, and overall, all carried a low risk of bias. In contrast, one study, the ACTIVE study by Lanzilo et al., has a high risk of bias with potential for selection bias, inadequate blinding as the study frame was open-label along with inadequate details to evaluate other potential areas of bias with the possibility of conflict of interest as the funds were raised by the pharmaceutical company and its role in research conduct was not explored adequately in the manuscript [23].

One study, a phase II multicenter randomized controlled trial, evaluated the use of simvastatin 80 mg monotherapy in patients with SPMS; the study was of good methodological quality where 140 patients with SPMS were followed for 24 months [26]. One study explored the effect of simvastatin 80 mg in patients with optic neuritis; the study was of good quality, but the number of patients involved in the trial was relatively small, 64, and the duration of follow-up was short, six months [25].

Summary of the Main Outcomes of the Review

Effect of statins use on MS disease relapse: Four studies in RRMS reported relapse rate as outcome variable [22-24,27]. No difference in relapse rate in patients taking statin & interferon combination in comparison to interferon alone was reported by good quality Sorensen et al. and Lanzilo et al. (ARIANNA ) studies having 307 and 145 participants [24,27]. The remaining two studies reported changes in relapse rate concerning statin use; Lanzilo et al., in the ACTIVE study, reported a relatively high risk of bias and a low risk of relapse per year of follow-up in the statin & interferon group [23]. In contrast, Birnbaum et al. trial showed a significantly higher risk of clinical relapse in the statin & interferon combination group than the placebo & interferon group [22]. However, the study is probably underpowered with a short duration of follow-up. Chataway et al. trial, the only trial conducted on SPMS, reported no effect of simvastatin monotherapy on disease relapse rate [26]. Overall, no adequate evidence of statin effect on disease relapse in RRMS and SPMS was concluded from the five included studies.

Effect of statins use on EDSS MS: Four studies in RRMS reported EDSS as outcome variables; three studies, Sorensen et al., ARIANNA studies, and Ghasami et al., reported no statin and interferon combination EDSS compared to placebo [24,27,28]. The ACTIVE study highlighted a significant non- trend towards better EDSS scores in statin interferon combination than in the interferon monotherapy group [23]. Chataway et al.'s trial concluded a significant positive outcome in EDSS in the simvastatin monotherapy group compared to placebo in patients with SPMS at 24 months of follow-up [26].

The Effect of Statins on MRI Changes

Three studies on RRMS (Sorensen et al., ARIANNA study, and Ghasami et al. studies) and Chataway et al.'s study on SPMS showed no effect of statin use on MRI changes [24,26-28]. The remaining two studies on RRMS showed contrasting effects on MRI lesions changes; Lanzilo in the ACTIVE study showed significantly lower MRI lesions in the treatment group, while Birnbaum et al. detected a higher risk of MRI reactivity in the intervention group [22,23].

Summary of the statins use outcomes in optic neuritis: Simvastatin used in optic neuritis showed significant benefits on the visual acuity, color perception, visual evoked potential (VEP) latency and VEP amplitude in simvastatin arm compared to placebo. However, there was no statistically significant difference detected in the total number of lesions in MRI or the rate of conversion to definite MS [25].

The beneficial role of simvastatin detected in SPMS, and optic neuritis could suggest that statin effects could be related to the level of activity of the disease and might support a role for simvastatin or other statins at the stages of the rapid inflammatory stage rather than the stable course seen in RRMS. More studies are currently warranted to examine these effects further and answer the logical question of whether these effects seen in RCTs are related to the whole class of statins or are only related to a particular statin medication (simvastatin).

Compared to previous reviews on statin effectiveness in MS, our review showed similar results to Wang et al., which concluded no clear evidence of statin's beneficial effect in patients with RRMS [16]. Our review has also suggested a positive effect of simvastatin in patients with SPMS similar to Pihl-Jensen et al.'s review [3]. Along with a trend towards positive effect in patients with optic neuritis, which needs to be replicated in more studies.

Potential sources of bias and the limitations of the review

Due to the small number of published RCTs on statin use in MS, only seven randomized control trials were included in our review. The review was limited to the English language published trials in five databases, while the results from unpublished literature and the rest of the databases were not explored. Most of the participating trials, except Sorensen et al., Lanzilo et al. ARIANNA study, and Chataway et al. studies, did not include a sample size calculation that raises the concern of being underpowered for the detection of treatment effect.

Furthermore, the review is also limited by the heterogeneity of the studies included; the trials covered different patient’s populations, various statins types and dosages used, and different formulations and doses of interferon used along with the variation in the total duration of follow-up, which was of the relatively short period between six to 36 months. All these factors complicate reaching a consistent conclusion.

## Conclusions

This systematic review aims to examine the effectiveness of statins in slowing the clinical and radiological course of MS. It showed that statins modification of MS progression previously expressed in the animal model and observational studies is not replicated in RCTs in patients with RRMS. However, there is evidence that simvastatin monotherapy might have beneficial outcomes on disability levels in patients with SPMS. Additionally, simvastatin use in optic neuritis showed a trend towards clinical improvement. In this review, we explored the role of affordable, well-tolerated oral medications in treating one of the disabling neurological disorders, MS, which has other treatment options with variable safety, efficacy, tolerability, and cost-effectiveness profile. We recommend performing more RCTs with sufficient patient numbers and a longer duration of follow-up either with statin alone or in combination with other approved medications for MS to examine further the effectiveness of statins on MS courses.
